# The influence of brain metastases on the central nervous system effects of methylnaltrexone: a post hoc analysis of 3 randomized, double-blind studies

**DOI:** 10.1007/s00520-021-06070-7

**Published:** 2021-02-25

**Authors:** Darren M. Brenner, Neal E. Slatkin, Nancy Stambler, Robert J. Israel, Paul H. Coluzzi

**Affiliations:** 1 Northwestern University Feinberg School of Medicine, Chicago, IL USA; 2 University of California, Riverside, School of Medicine, Riverside, CA USA; 3 Salix Pharmaceuticals, Bridgewater, NJ USA; 4 Progenics Pharmaceuticals, Inc., a subsidiary of Lantheus Holdings, Inc., New York, NY USA; 5 Bausch Health US, LLC, Bridgewater, NJ USA; 6 UCI Health, Orange, CA USA

**Keywords:** Methylnaltrexone, Narcotic antagonists, Analgesics, Opioid, Constipation, Pain management

## Abstract

**Purpose:**

Peripherally acting μ-opioid receptor antagonists such as methylnaltrexone (MNTX, Relistor^®^) are indicated for the treatment of opioid-induced constipation (OIC). The structural properties unique to MNTX restrict it from traversing the blood-brain barrier (BBB); however, the BBB may become more permeable in patients with brain metastases. We investigated whether the presence of brain metastases in cancer patients compromises the central effects of opioids among patients receiving MNTX for OIC.

**Methods:**

This post hoc analysis of pooled data from 3 randomized, placebo-controlled trials included cancer patients with OIC who received MNTX or placebo. Endpoints included changes from baseline in pain scores, rescue-free laxation (RFL) within 4 or 24 h of the first dose, and treatment-emergent adverse events (TEAEs), including those potentially related to opioid withdrawal symptoms.

**Results:**

Among 356 cancer patients in the pooled population, 47 (MNTX *n* = 27; placebo *n* = 20) had brain metastases and 309 (MNTX *n* = 172; placebo *n* = 137) did not have brain metastases. No significant differences in current pain, worst pain, or change in pain scores from baseline were observed between patients treated with MNTX or placebo. Among patients with brain metastases, a significantly greater proportion of patients who received MNTX versus placebo achieved an RFL within 4 h after the first dose (70.4% vs 15.0%, respectively, *p* = 0.0002). TEAEs were similar between treatment groups and were generally gastrointestinal in nature and not related to opioid withdrawal.

**Conclusion:**

Focal disruptions of the BBB caused by brain metastases did not appear to alter central nervous system penetrance of MNTX.

## Introduction

When conservative pharmacologic measures are inadequate to treat cancer-related pain, opioid treatment should be considered. Opioids activate the endogenous pain-modulating system through agonism of several opioid receptor types such as μ-, κ-, and δ-opioid receptors in the central nervous system [1]. μ-Opioid receptors are also located in the gastrointestinal tract, where their activation can lead to reductions in motility and fluid secretion, and increased fluid reabsorption resulting in increased transit time [2]. As a result, opioid-induced constipation (OIC), a subset of the broader opioid-induced bowel disorders spectrum of symptoms [3], is the most common side effect of opioid-based analgesia regimens and can affect the majority of patients treated with chronic opioid therapy [4–7].

The blood-brain barrier (BBB) comprises endothelial cells interconnected by tight junctions that line capillaries within the brain and allows the selective, highly regulated diffusion of specific molecules into the brain via facilitated or active transport [8]. Restricted diffusion of compounds across the BBB is due, in part, to structural and biochemical features such as high molecular weight, large molecular volume, or hydrophilicity [9–14]. A class of peripherally acting μ-opioid receptor antagonists (PAMORAs) was developed for the treatment of OIC that act by blocking opioid binding to μ-opioid receptors in the gastrointestinal tract. PAMORAs have specific design features (e.g., low lipid solubility, large structure, strong polarity) that reduce their penetrance through the BBB, thereby minimizing effects on centrally mediated opioid analgesia and preventing opioid withdrawal [15–17].

In patients with brain metastases, changes in vasculature within brain tumors can lead to loss of tight junctions and increased fenestrations in endothelial cells, compromising the biochemical and structural integrity of the BBB [8, 18]. Hypothetically, if this barrier is disrupted, it is possible for patients who use opioid inhibitors to reduce OIC to be at increased risk of centrally mediated effects of opioid withdrawal. In fact, product labeling for drugs in the PAMORA class warns that the overall risk benefit in patients with disruptions to the BBB must be considered and patients should be monitored closely for symptoms of opioid withdrawal and/or reduced analgesia [15–17].

Methylnaltrexone (MNTX; Relistor^®^, Salix Pharmaceuticals, a division of Bausch Health US, LLC, Bridgewater, NJ, USA) is a PAMORA that is similar in structure to naltrexone, but is methylated to form a quaternary amine which, due to its polarity, is much less likely to diffuse across the BBB [19–21]. MNTX has been shown to decrease the constipating effect of opioid therapy without compromising analgesia or precipitating symptoms of opioid withdrawal [16, 22–24]. MNTX tablets and subcutaneous (SC) injection are approved for the treatment of OIC in adults with chronic noncancer pain, including those with inactive cancer pain who do not require opioid dose increases. SC MNTX is also approved to treat OIC among patients with advanced illness or active cancer being palliated for chronic pain [16]. Although labeling for MNTX and other agents in its class warns that use in individuals with disruptions to the BBB may precipitate symptoms of opioid withdrawal and reduced analgesia, no previous studies have specifically examined whether conditions associated with increased BBB permeability have led to clinical signs of opioid withdrawal after PAMORA use. Therefore, it is unknown if these changes to the BBB truly impact the central nervous system penetrance of MNTX. This post hoc analysis of pooled data examined a subset of cancer patients with brain metastases who received MNTX or placebo for OIC to determine if the central analgesic effects of opioids were compromised by the presence of brain metastases.

## Methods

### Study design

This was a pooled, post hoc analysis based on 3 multicenter, double-blind, randomized, placebo-controlled trials in adult patients with advanced illness, including patients with active cancer and OIC, the primary results of which have been published [23–25]. Each study site obtained institutional review board and independent ethics committee approval for the protocols and informed consent forms. Each study was conducted in compliance with the principles of Good Clinical Practice and in accordance with the Declaration of Helsinki. All study participants provided written informed consent. In study 301 [NCT00401362] [23], following a 5-day screening period, patients were randomized 1:1:1 to receive single SC injections of MNTX 0.15 mg/kg (*n* = 47), MNTX 0.30 mg/kg (*n* = 55), or placebo (*n* = 52). In study 302 [NCT00402038] [24], patients were randomized to receive SC injections of MNTX 0.15 mg/kg (*n* = 63) or placebo (*n* = 71) every other day for 2 weeks. The dose could be adjusted up to 0.30 mg/kg beginning on day 9. In study 4000 [NCT00672477] [25], patients received MNTX or placebo every other day for a maximum of 7 doses for 14 days; the dose of MNTX was determined by body weight. Patients weighing 38 to < 62 kg were randomized to receive SC injections of MNTX 8 mg or placebo (*n* = 45), while patients weighing ≥ 62 kg were randomized to receive SC injections of MNTX 12 mg (*n* = 71) or placebo (*n* = 114).

OIC was defined as < 3 bowel movements during the previous week and no clinically significant laxation during the 24 h (studies 302 and 4000) or 48 h (study 301) preceding the first dose of study drug. If the patient was receiving a laxative (e.g., stool softener and senna or equivalent), the regimen had to be stable for ≥ 3 days prior to the first dose of study drug. For all studies, rescue laxatives and enemas were permitted but not within 4 h before or after study drug administration. Rescue doses of opioids were permitted as necessary.

### Patients

For eligibility in the pooled analysis, men and women were required to be ≥ 18 years of age, with a life expectancy of ≥ 1 month (studies 302 and 4000) or 1 to 6 months (study 301); receiving opioids routinely for discomfort or pain management for ≥ 3 days (study 301) or ≥ 2 weeks (studies 302 and 4000), excluding as needed or rescue doses and taking a stable regimen for ≥ 3 days before the first dose of study medication. A stable regimen was defined as no reduction of ≥ 50% in opioid dose within 3 days prior to study drug administration.

Candidates were excluded from the study if they had a prior history of MNTX treatment (except in study 4000 where MNTX was allowed before the 7-day washout period), any disease process suggestive of mechanical bowel obstruction, evidence of fecal impaction, history of fecal ostomy, or any potential nonopioid cause of bowel dysfunction that, in the opinion of the investigator, may have been primarily responsible for the constipation.

### Assessments

Baseline characteristics, including the baseline morphine equivalent dose, were collected. For the purposes of this analysis, baseline demographics and safety data were reported for cancer patients with brain metastases who received MNTX or placebo and for cancer patients without brain metastases who received MNTX or placebo. Efficacy outcomes (pain intensity and rescue-free laxation [RFL] response within 4 and 24 h after the first dose) were analyzed to compare patients with brain metastases who received MNTX versus placebo. Changes in pain score from baseline to 4 h after the first dose (both current pain and worst pain since baseline) were measured using a patient-reported rating scale of 0 (no pain) to 10 (worst possible pain).

Safety assessments included treatment-emergent adverse events (TEAEs) reported on treatment day 1 and treatment day 2. TEAEs potentially related to opioid withdrawal symptoms were collected by identifying MedDRA-defined TEAEs that are described on the modified Subjective Opioid Withdrawal Scale (SOWs). The SOWs has been used in previous MNTX studies as a patient-rated measurement of the severity of opioid withdrawal symptoms [26, 27]. The scale has 16 questions that rates patients’ perceived severity of opioid withdrawal symptoms on a scale from 0 (not at all) to 4 (extremely) [28]. Our scale was modified to include 3 additional items (trouble sleeping, poor appetite, and diarrhea) to account for a population of patients with OIC, for a total possible score of 76.

### Statistical analyses

Baseline characteristics were assessed using descriptive statistics. For the analysis of pain scores, *p* values were based on *t* tests to compare the MNTX and placebo groups. Analyses of RFL response and change from baseline for pain scores were performed on the intent-to-treat analysis set, defined as all patients who received at least 1 dose of study drug. Patients who reported an RFL within the first 4 or 24 h were considered responders. *p* values for an RFL response within 4 and 24 h were based on chi-squared tests. Nominal levels of significance were set at 0.05, with no adjustment made for multiplicity. Safety data were assessed among the safety population, which included all randomized patients.

## Results

### Patients

**Fig. 1:**
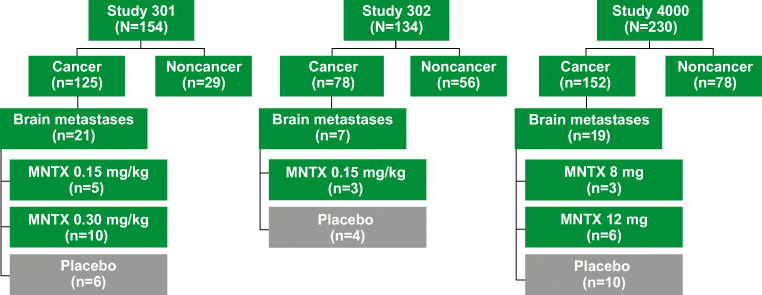
Disposition of patients by study and by those with brain metastases based on treatment group. MNTX methylnaltrexone

When pooled, there were 518 patients randomized in the 3 studies (study 301 = 154; study 302 = 134; study 4000 = 230; Fig. 1). In this advanced illness pooled population, 356 (69%) had a cancer diagnosis at baseline from which 47 (*n* = 27 MNTX; *n* = 20 placebo) had brain metastases and 309 (*n* = 172 MNTX; *n* = 137 placebo) did not have brain metastases (Fig. 1). Baseline characteristics among cancer patients with brain metastases who received MNTX or placebo were similar (Table 1). Median opioid morphine equivalent daily doses were 180.0 mg/day and 225.0 mg/day among patients with brain metastases who received MNTX or placebo, respectively. More patients with brain metastases receiving MNTX used corticosteroids than those receiving placebo (MNTX 70.4%; placebo 55.0%), and most patients used 1 to 3 laxatives at baseline.

**Table 1:** Patient baseline characteristics by double-blind treatment and presence of brain metastasis among cancer patients (pooled safety population^a^)

	Patients with brain metastases		Patients without brain metastases	
	MNTX (*n* = 27)	Placebo (*n* = 20)	MNTX (*n* = 172)	Placebo (*n* = 137)
Age (years), mean (range)	62.9 (44, 87)	60.0 (41, 83)	63.7 (26, 91)	64.4 (21, 100)
Gender, *n* (%)				
Male	12 (44.4)	8 (40.0)	96 (55.8)	73 (53.3)
Female	15 (55.6)	12 (60.0)	76 (44.2)	64 (46.7)
Race, *n* (%)				
White	23 (85.2)	17 (85.0)	151 (87.8)	122 (89.1)
Black/African American	2 (7.4)	0	11 (6.4)	9 (6.6)
Asian	1 (3.7)	1 (5.0)	1 (0.6)	0
Hispanic	1 (3.7)	1 (5.0)	6 (3.5)	4 (2.9)
Other	0	1 (5.0)	3 (1.7)	2 (1.5)
Weight (kg), mean (range)	69.6 (32.7, 110.0)	68.8 (49.4, 93.7)	69.9 (30.9, 135.8)	70.2 (29.0, 138.0)
Daily dose opioid morphine equivalent (mg/day)				
Mean ± SD	470.8 ± 816.6	308.9 ± 313.0	1461.4 ± 9695.1	609.9 ± 1489.8
Median (range)	180.0 (45.0, 3920.0)	225.0 (20.0, 1140.0)	195.0 (0, 122,560.0)	190.7 (0, 10,160.0)
Use of corticosteroids, *n* (%)	19 (70.4)	11 (55.0)	62 (36.0)	55 (40.1)
Number of laxatives used at baseline, *n* (%)				
0	0	1 (5.0)	7 (4.1)	1 (0.7)
1	11 (40.7)	5 (25.0)	51 (29.7)	34 (24.8)
2	10 (37.0)	10 (50.0)	66 (38.4)	46 (33.6)
3	4 (14.8)	4 (20.0)	25 (14.5)	31 (22.6)
4	2 (7.4)	0	17 (9.9)	18 (13.1)
5	0	0	5 (2.9)	3 (2.2)
6	0	0	1 (0.6)	3 (2.2)
7	0	0	0	1 (0.7)
Baseline current pain score, mean ± SD	3.4 ± 3.0	3.6 ± 2.8	3.4 ± 2.6	3.4 ± 2.5
Baseline worst pain score, mean ± SD	5.1 ± 2.9	5.1 ± 3.2	5.3 ± 2.8	5.3 ± 2.7

*MNTX* methylnaltrexone, *SD* standard deviation

^a^The safety population includes all randomized patients who received ≥ 1 dose of study drug

Patients without brain metastases were slightly older and were represented by more men than those with brain metastases. The median daily dose of opioid morphine equivalents used by patients without brain metastases was similar to the dose used by patients with brain metastasis while fewer patients without brain metastases used corticosteroids. Baseline pain scores were similar between treatment groups among patients with and without brain metastases (Table 1). No patient included in the analysis had previous exposure to MNTX. The most frequently reported primary cancers were breast and lung cancer (Table 2).

**Table 2:** Primary cancers at baseline (pooled safety population)^a^

	MNTX (*n* = 27)	Placebo (*n* = 20)
Lung	10	6
Breast	5	5
Unknown primary tumor	3	0
Ovarian	2	0
Prostate	2	1
Cervical	1	0
Renal	1	2
Esophageal	1	0
Melanoma	1	3
Adenocarcinoma	1	0
Rectal	0	1
Gastrointestinal stromal tumor	0	1
Pancreatic	0	1

*MNTX* methylnaltrexone

^a^The safety population includes all randomized patients who received ≥ 1 dose of study drug

### Pain scores after the first dose

**Fig. 2:**
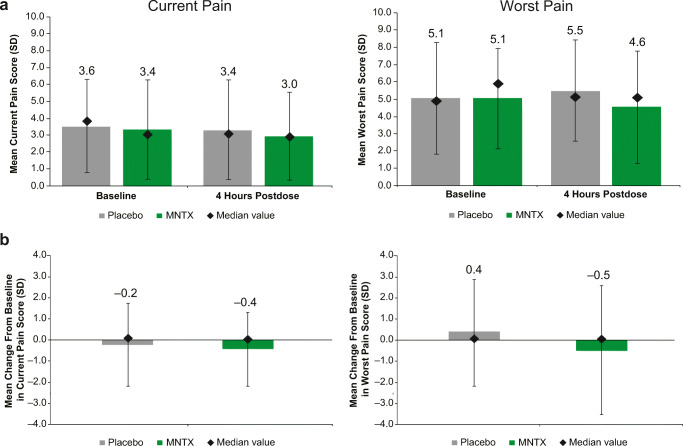
Pain scores after administration of placebo or MNTX among those patients with brain metastases. **a** Current and worst pain scores at baseline and 4 h after the first dose and **b** change from baseline in current and worst pain scores at baseline and 4 h after the first dose (pooled safety population [ITT]; the safety population includes all randomized patients who received ≥ 1 dose of study drug). MNTX methylnaltrexone, ITT intent to treat, SD standard deviation. Pain was scored as 0 = no pain to 10 = worst possible pain. Diamonds represent median values; error bars represent standard deviation

We compared differences in pain scores between patients with brain metastases treated with MNTX or placebo at both baseline and 4 h after treatment. No significant differences occurred between treatment groups in either current pain or worst pain 4 h after the first dose (Fig. 2a). Mean ± SD current pain scores in patients treated with MNTX or placebo were 3.0 ± 2.65 and 3.4 ± 3.13, respectively, 4 h after treatment (*p* = 0.3257), representing changes from baseline of − 0.4 and − 0.2 (*p* = 0.3439; Fig. 2b). Mean ± SD worst pain scores in patients treated with MNTX or placebo were 4.6 ± 3.26 and 5.5 ± 2.64, respectively, 4 h after treatment (*p* = 0.3257; Fig. 2a), representing changes from baseline of − 0.5 and 0.4 (*p* = 0.3439; Fig. 2b).

### Rescue-free laxation response within 4 h after the first dose

**Fig. 3:**
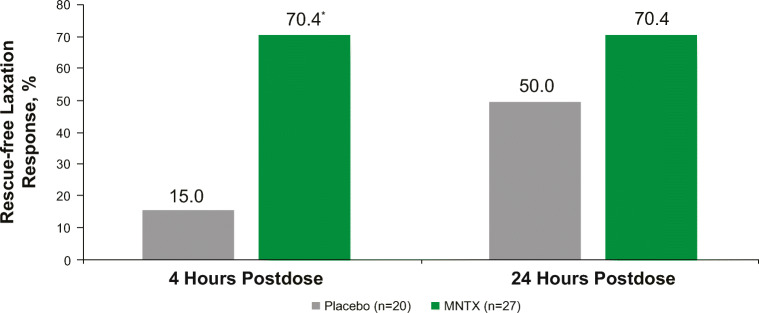
Rescue-free laxation response within 4 h or 24 h after the first dose among patients with brain metastases. *p = 0.0002 vs placebo

Among patients with brain metastases, a significantly greater proportion of those who received MNTX than placebo achieved an RFL response within 4 h after the first dose (70.4% vs 15.0%, *p* = 0.0002; Fig. 3). After 24 h, the proportion of patients achieving a response was the same as at 4 h for the MNTX-treated group (70.4%) but increased to 50.0% of patients who received placebo (*p* = 0.1555).

### Adverse events

Safety results are summarized in Tables 3 and 4. The percent of patients with at least 1 TEAE was 48.1% on treatment day 1 and declined to 36.4% on treatment day 2 among patients with brain metastases who received MNTX. Although this percentage was higher when compared with the placebo group (placebo 15.0% on treatment day 1 and 16.7% on treatment day 2), the difference was largely attributed to the incidence of abdominal pain reported in the MNTX group (Table 3).

**Table 3:** Most common TEAEs on treatment day 1 and day 2 in patients with and without brain metastases (> 5% in any treatment group in the double-blind studies; pooled safety population^a^)

Preferred term, *n* (%)	Patients with brain metastases				Patients without brain metastases			
	Placebo		MNTX		Placebo		MNTX	
	Day 1 *n* = 20	Day 2 *n* = 12	Day 1 *n* = 27	Day 2 *n* = 11	Day 1 *n* = 137	Day 2 *n* = 90	Day 1 *n* = 172	Day 2 *n* = 93
Patients with at least 1 TEAE	3 (15.0)	2 (16.7)	13 (48.1)	4 (36.4)	33 (24.1)	13 (14.4)	78 (45.3)	23 (24.7)
Abdominal pain^b^	0	0	6 (22.2)	2 (18.2)	7 (5.1)	5 (5.5)	39 (22.7)	8 (8.6)
Nausea	0	0	2 (7.4)	0	1 (0.7)	1 (1.1)	9 (5.2)	3 (3.2)
Flatulence	0	0	1 (3.7)	0	5 (3.6)	2 (2.2)	12 (7.0)	2 (2.2)
Pain exacerbated	0	0	2 (7.4)	0	3 (2.2)	1 (1.1)	4 (2.3)	0
Muscle cramp	0	0	1 (3.7)	1 (9.1)	0	0	1 (0.6)	0
Muscle rigidity	0	1 (8.3)	0	0	0	0	0	0
Dizziness	0	0	0	1 (9.1)	0	0	7 (4.1)	0
Headache	0	0	0	1 (9.1)	1 (0.7)	0	3 (1.7)	0
Dyspnea exacerbated	0	1 (8.3)	0	0	0	0	0	0
Pruritus	0	0	0	1 (9.1)	0	0	0	1 (1.1)

*MNTX* methylnaltrexone, *TEAE* treatment-emergent adverse event

^a^The safety population includes all randomized patients who received ≥ 1 dose of study drug

^b^Includes abdominal pain and abdominal pain not otherwise specified

**Table 4:** Cancer patients with and without brain metastases who experienced TEAEs potentially related to opioid withdrawal (> 5% in any treatment group in the double-blind studies; pooled safety population^a^)

	Patients with brain metastases		Patients without brain metastases	
Preferred term, *n* (%)	MNTX (*n* = 27)	Placebo (*n* = 20)	MNTX (*n* = 172)	Placebo (*n* = 137)
Patients with ≥ 1 TEAEpotentially related to OW	17 (63.0)	9 (45.0)	102 (59.3)	68 (49.6)
Nausea	9 (33.3)	2 (10.0)	27 (15.7)	18 (13.1)
Vomiting^b^	4 (14.8)	4 (20.0)	23 (13.4)	20 (9.5)
Sweating increased	4 (14.8)	0	13 (7.6)	11 (8.0)
Diarrhea^c^	4 (14.8)	1 (5.0)	14 (8.1)	12 (8.8)
Restlessness	3 (11.1)	1 (5.0)	11 (6.4)	10 (7.3)
Agitation	3 (11.1)	0	11 (6.4)	8 (5.8)
Anxiety	3 (11.1)	0	13 (7.6)	12 (8.8)
Abdominal pain	2 (7.4)	1 (5.0)	26 (15.1)	10 (7.3)
Rhinorrhea	1 (3.7)	0	12 (7.0)	3 (2.2)
Arthralgia	1 (3.7)	2 (10.0)	10 (3.3)	3 (1.4)
Insomnia	1 (3.7)	0	8 (4.7)	7 (5.1)
Tremor	0	0	8 (4.7)	7 (5.1)

*MNTX* methylnaltrexone, *OW* opioid withdrawal, *TEAE* treatment-emergent adverse event

^a^The safety population includes all randomized patients who received ≥1 dose of study drug

^b^Includes vomiting and vomiting not otherwise specified

^c^Includes diarrhea and diarrhea not otherwise specified

The incidences of TEAEs that correspond to symptoms assessed on the SOWs and could potentially be related to opioid withdrawal are presented in Table 4. The presence of brain metastases did not impact the percentage of patients with at least 1 TEAE potentially related to opioid withdrawal (patients with brain metastasis: placebo, 45.0% vs MNTX, 63.0%; patients without brain metastasis: placebo, 49.6% vs MNTX, 59.3%).

## Discussion

The BBB is a protective physiologic barrier that serves to prevent substances from entering vital areas of the brain. Some drugs such as the opioid antagonist naltrexone have a lipophilic structure that allows them to readily penetrate the BBB and to exert centrally mediated inhibition of opioid receptors [29]. In contrast, MNTX, a quaternary amine of naltrexone, was designed to restrict its access to the brain while preserving antagonism at peripheral μ-opioid receptors in the gastrointestinal tract [19–21]. Clinical studies have demonstrated that MNTX does not traverse the intact BBB as evidenced by the absence of increases in pain intensity or other signs of opioid withdrawal following MNTX treatment [23–25].

This retrospective analysis of 3 double-blind, placebo-controlled studies of patients receiving opioid therapy revealed that the use of MNTX effectively reduced the occurrence of OIC but did not lead to increases in pain or symptoms of opioid withdrawal in a cohort of patients with brain metastases. Worsening of pain is a sensitive and early indicator of opioid withdrawal in patients with chronic pain receiving opioids [30], and in our study, pain levels did not significantly differ from baseline in patients treated with either MNTX or placebo with observed changes actually indicating a small mean reduction in pain from baseline levels in MNTX-treated individuals. Moreover, no significant differences in TEAEs potentially related to opioid withdrawal were observed when comparing patients treated with MNTX versus placebo.

The studies that provided the data for our pooled analysis revealed that MNTX was effective in facilitating laxation in patients treated with opioids to manage pain for a range of terminal advanced medical illnesses. Specifically, MNTX led to significant increases in the proportions of patients who achieved RFL at 4 and 24 h compared with placebo, with RFL response differentials of 33 to 48% and 41 to 44% at each time point, respectively [23, 24]. In our subset analysis in patients with brain metastases, RFL response at 24 h after the first dose of MNTX was similar but, with an RFL response differential of 20.4%, did not reach statistical significance compared with the placebo group. However, the proportion of MNTX-treated patients with brain metastases who achieved RFL at 4 h in the current analyses exceeded the proportions observed in the previous studies and was significantly greater than in the placebo group. The reduced effect at 24 h in our study compared with the prior studies may be attributed to the small sample size of patients with brain metastases, although we cannot rule out that some other unknown factors related to brain metastases may have influenced this result. Nonetheless, the significant results from the original studies coupled with the current analysis demonstrate that MNTX can effectively antagonize μ-opioid receptors in the periphery to facilitate laxation in patients with brain metastases.

Although the original studies assessed laxation response, the key question in the current analysis was whether brain metastases result in changes that allow MNTX to breach the BBB and affect the centrally located μ-opioid receptors targeted for pain management. Pain levels remained unchanged as indicated by the lack of significant changes from baseline observed between the groups. Clinically meaningful differences in pain intensity are considered when pain scores change by 2 points in populations with chronic low back pain [31]. However, it is unclear what threshold equates to clinically meaningful differences in cancer patients. In our analysis, the mean changes were less than 1 point, which further supports the finding that opioid analgesia was not affected by brain metastases.

The overall pattern of adverse events related to opioid withdrawal in our study was similar between cancer patients with and without brain metastases, suggesting that any change in BBB permeability possibly caused by brain metastases did not result in a significant reduction in analgesia, nor did it result in signs of opioid withdrawal. Further, the most prevalent TEAEs that could potentially be related to opioid withdrawal were nausea, vomiting, and diarrhea, which are also common during normal laxation. The most frequently reported adverse events on treatment day 1 and treatment day 2 in MNTX-treated patients with and without brain metastases were abdominal pain, nausea, and flatulence. These effects declined by treatment day 2, and as such, can reasonably be attributed to effective laxation.

There is evolving research on the influence of μ-receptor antagonism on all-cause mortality and cancer progression [32–41]. Preclinical studies have suggested that μ-receptor activation may promote tumor progression via angiogenesis or through mediation of cellular processes important for tumor growth [42–44]. From a clinical perspective, the impact of MNTX treatment on patient survival was analyzed in 2 clinical trials of patients with advanced cancers. In a combined analysis of the 2 studies, MNTX use significantly prolonged survival compared with placebo (76 vs 56 days, *p* = 0.033). The authors speculated that the mechanism may have direct effects on gut function or possibly an indirect effect on immunosuppression [41]. For ongoing MNTX studies in patients with advanced cancer, it is of importance to know that MNTX efficacy and safety is not impacted by brain lesions.

There were several limitations to our study that should be considered. First, this was a retrospective, post hoc analysis examining a small subset of patients from 3 larger studies. As a result, we were limited to a small population of patients, and the analysis was not powered for effective statistical comparisons. This analysis only assessed the effects of MNTX on OIC for an acute period of time (up to 24 h); longer observation periods may be useful to see the longer-term effects of brain metastases on central analgesia among patients taking MNTX. Moreover, the designs of the original studies varied slightly, including different dosages of MNTX and slight differences in the definitions of OIC and in inclusion/exclusion criteria. It is unknown if these differences may have impacted the results. However, it should be noted that the efficacy and safety results of this post hoc analysis were similar to those observed in the overall studies. Patients with brain metastases were identified post hoc based on a retrospective review of patient narratives. Since the double-blind period of the pooled studies were of short duration, it is likely that the majority of patients in this subanalysis had metastases at baseline. None of the patients in this series had a primary brain tumor, and the exact location of the brain metastases was not recorded.

In conclusion, this analysis demonstrated that in patients with brain metastases—a condition likely to increase permeability of the BBB—MNTX did not lead to any significant changes in pain or opioid withdrawal symptoms yet reduced the occurrence of OIC. This suggests that MNTX is safe and effective in these patients. Hence, focal disruptions in the BBB caused by brain metastases may not sufficiently alter the penetrance of MNTX across the BBB to the extent that might cause clinical concern.

## Data Availability

The datasets generated and/or analyzed during the current study are not publicly available at this time due to the proprietary nature of this information. Requests for additional information should be made to the corresponding author.
